# Sensor Calibration Based on Incoherent Optical Fiber Bundles (IOFB) Used For Remote Image Transmission

**DOI:** 10.3390/s91008215

**Published:** 2009-10-19

**Authors:** José L. Lázaro, Pedro R. Fernández, Alfredo Gardel, Angel E. Cano, Carlos A. Luna

**Affiliations:** 1 Electronics Department, University of Alcalá, Superior Polytechnic School, Universitary Campus, Alcalá de Henares (28871), Madrid, Spain; E-Mails: pedro.fernandez@depeca.uah.es (P.R.F.); alfredo@depeca.uah.es (A.G.); caluna@depeca.uah.es (C.A.L.); 2 Telecommunication Department, University of Oriente, Av. de las Américas, SN, Santiago de Cuba (90900), Cuba; E-Mail: angel.cano@depeca.uah.es

**Keywords:** image sensor, image transmission, sensor calibration, optical fiber sensors

## Abstract

Image transmission using incoherent optical fiber bundles (IOFB) requires prior calibration to obtain the spatial in-out fiber correspondence in order to reconstruct the image captured by the pseudo-sensor. This information is recorded in a Look-Up Table (LUT), used later for reordering the fiber positions and reconstructing the original image. This paper presents a method based on line-scan to obtain the in-out correspondence. The results demonstrate that this technique yields a remarkable reduction in processing time and increased image quality by introducing a fiber detection algorithm, an intensity compensation process and finally, a single interpolation algorithm.

## 1. Introduction

Current visual inspection systems based on camera arrays are widely applied. However, these systems are not suitable for smooth surfaces or in environments with adverse conditions where the use of electrical signals or electronic devices can be problematic, such as where there is a risk of explosion, in high-temperature ovens, medical endoscopies, periscopes, nuclear reactors, or places far from the control unit. Under these conditions, the use of optical fiber bundles coupled with a CMOS sensor could be advantageous, isolating the environment under inspection from the sensor [1-10]. Any image projected onto the entrance plane is broken down at the opposite end into multiple individual spots (one per fiber). Generally, IOFBs are used for imaging purposes because the image formed at the end preserves its initial aspect.

Nevertheless, despite the non spatial correspondence of the fibers between both ends, an incoherent optical fiber bundle (IOFB) can be used for the same purpose. Nowadays, coherent bundles are used for image transmission over relatively short distances in medical endoscope equipment, fiberscopes, or videoscopes. To reduce interstitial space between fibers, a fusion mechanism is applied to the bundle. As a result, the flexibility of the bundles is reduced and the possibility of fracturing the fibers increases. Therefore, the length of the bundle used varies from a few centimeters to a meter, and cost rises with increasing length and number of fibers [3].

IOFB fiber layout does not need to conform to any specific configuration; hence, the manufacturing process does not require great precision and samples can be obtained without length restrictions and at a lower cost than coherent bundles. To transmit the image correctly, a calibration process is essential in order to retrieve the in-out correspondence for a posterior reconstruction of the captured image. In addition to being flexible and robust, this mechanism permits the use of relatively low-priced fiber bundles of almost unlimited length. The main drawback of these systems is that the quality of the reproduction image is lower when compared to coherent bundles. Image quality is affected primarily by non-uniform fiber distribution and the existence of areas with large spaces between fibers or the absence of fibers.

In an earlier paper [11], the authors presented a brief introduction to the location of fibers in the bundle. This process enabled the individual response of the fibers to be obtained in order to reduce time spent in calibration and to improve the intensity compensation of the final image. In this paper we describe the entire calibration process using a line-scan method (multifiber). Some qualitative results for calibration are presented, taking into account the influence of different parameters in image reconstruction.

## 2. Background

### 2.1. System Setup

**Figure 1.**
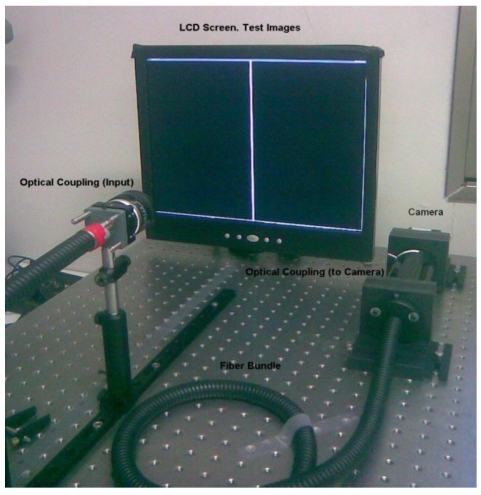
System setup.

Figure 1 shows the setup for implementing calibration. It comprises a LCD screen, a flexible fiber bundle coupled, in the input face, with a suitable optic for projecting the images generated onto the screen, and at the other face with another optic coupling to a CMOS camera (gray scale). It should be emphasized that this fiber bundle is designed for illumination purposes and is not, therefore, optimized for image transmission applications.

Each image (generated from a PC to a LCD monitor), describes a line, the position of which varies at each step, scanning the input face of the bundle through the input optics. A given number of fibers are illuminated at each step describing a disperse constellation of excited fibers at the output side of the guide, which are focused with a CMOS camera using another optic coupling.

By exciting the input side of the bundle with the test images, it is possible to obtain the input-output correspondence of fiber distribution using the sequential images captured by the CMOS sensor and a suitable image processing algorithm. A sequential variation of these input images across all of the fibers allows in-out calibration, locating those pixels which were notably excited (illuminated in the output side). The obtained correspondence is recorded on an LUT (Look-up table).

The following IOFB characteristics should be taken into account:

1. A different input-output transference ratio between the fibers.
2. Most fibers (not broken or deformed) are the same size.
3. Fibers are circular and are separated by a small space.
4. There are some areas where a partial absence of fibers and a non-uniform distribution of fibers can be found.
5. Only a few fibers are deformed by a superficial flaw.

Under these conditions, it should be borne in mind that any image captured at the entrance of the bundle not only comes out disorganized but also with important changes to local intensities, due to non-uniform fiber distribution, the relationship between the input optic and the fiber aperture, and the presence of diverse transference functions in the fibers. These characteristics, and particularly the first, obliged us to particularize the behavior of each fiber in the bundle, in order to ensure a certain level of reconstructed image quality.

### 2.2. Previous Research

All existing methods for calibrating IOFBs employ a unique principle which consists in projecting a specific number of known pattern images, and verifying the fibers on the outside that reach a considerable level of energy. Early attempts used a moving spot on the calibration screen, but the time taken for the calculation was too high. In [2,3,6,10,11] the use of multi-fiber excitation techniques for calibration has been suggested. These techniques enable an indeterminate number of fibers to be excited at the same time, reducing calibration time significantly. In [2,6], the authors employed a group of codified images comprising stripes of different sizes. This method is rapid and requires very few images; however, calculation of LUT is quite complicated.

In [3,11], a line scanning principle is suggested. This permits multi-fiber excitation and simplifies LUT calculations. The number of images needed is dependent on the number of fibers in the bundle and consequently, the quantity of images to be verified is greater than in [2,6]. Nowadays, this does not present a problem.

In order to reconstruct and equalize the image, it was necessary to determine individual fiber features (location, intensity response), and this was achieved through the use of the FDDT (Fiber detection by Distance Transform) method. This guarantees a high detection rate for elements, a highly acceptable processing speed and the ability to work with smaller image resolutions (*nxn* pixels per fiber) [11].

A significant reduction in the processing time is obtained by an a priori knowledge of the real positions of the fiber cores, as only a limited number of coordinates in the sensor are analyzed during the calibration process, reducing the number of operations, the memory storage requirements and data processing time. Note that for each image *I*, it is necessary to verify whether any of the thousands of fibers comprising the bundle are sufficiently excited. Only one pixel per fiber (feature point or characteristic pixel) is stored on the LUT. Because the reconstruction of the final image only depends on the information provided by the proximity of the characteristic points (a *kxk* windows) which have been pre-located, the image quality of the transmission system is greatly increased and consequently, detection of individual fibers is excellent. The gray level *g_i_* contributed by each *i* fiber is:

(1) $$\begin{array}{l} {\text{g}}_{\text{i}}=\frac{\text{1}}{\text{k}\times \text{k}}\sum_{\text{x}=x_{ci}-\frac{k-1}{2}}^{{\text{x}}_{\text{ci}}+\frac{k-1}{2}}\sum_{\text{y}={\text{y}}_{\text{ci}}-\frac{k-1}{2}}^{{\text{y}}_{\text{ci}}+\frac{k-1}{2}}\text{I}(\text{x},\text{y})\\ g_{i}\subset I \end{array}$$

where *k* is an odd value, generally *k* = 3, and the coordinate pair (*x_c_,y_c_*) is the centroid of the *i* fiber on *I* image.

A further advantage is the possibility of modifying each fiber's response in order to equalize the g_i_ value. This is of great importance during the calibration process in order to ensure that each fiber produces a similar response to the same stimulus, thus reducing the differences of the transfer function and compensating the gray level individually. This is critical where the sensor has captured a homogeneous image or an image with smooth surfaces.

In FDDT fiber edge detection is not required which constitutes a significant advantage with respect to other circle detection algorithms such as Circular Hough Transform. During the first step, the image is subjected to a thresholding process in order to reset (in black) the intensity values of background and interstitial spaces. For an image *I* the size of NxM, an image binary BW can be obtained so that each pixel is:

(2) $$BW\left(x,y\right)=\left\{ \begin{array}{l} 1\to I(x,y)\geq \tau \\ 0\to I(x,y)<\tau \end{array}\right.$$

where the τ value is an optimal threshold of intensity obtained by the Otsu method:

(3) $$\tau =\underset{t}{\text{Max}}\left\{{\sigma_{B}}^{2}(t)\right\}\;1\leq t\leq L$$

where σ^2^ is the variance of classes in a thresholded image with *L* possible values of gray.

Through a double morphological mechanism of erosion and dilation for binary images, partial segmentation is obtained. Erosion and dilation should be carried out using a small structuring disk element *H*, since this may exclude several small fibers from the detection process.

(4) $$\begin{array}{l} \text{E}(\text{I})=\text{I}\Theta \text{H}=\{p|H_{p}\subseteq I\}\\ \text{D}(\text{I})=\text{I}⊕\text{H}=\underset{{\text{h}}_{\text{i}}\in \text{H}}{\cup I_{h_{i}}} \end{array}$$

The next step applies the Distance Transform (DT) to the inverted image of *BW*. DT provides a measurement of the separation between points in the image. Distance transformation is used to convert a digital binary image, consisting of object (foreground) and non-object (background) pixels, into another image in which the value of each object pixel corresponds to the minimum distance, as defined by a distance function, from the background. DT converts this binary image into a gray level image, where each pixel that was set to off (0) is assigned a number (gray level) which is the distance between a pixel and the nearest nonzero pixel (1). If there are two points *p(x,y)* and *q(u,v)* with different values, the distance function can be defined by:

(5) $$d_{ch}(p,q)=\max(\left|x-u\right|,\left|y-v\right|)$$

This is known as the chessboard metric. In general, DT affects the whole image and could represent a time-consuming process; however the process is carried out on a binary image with small closed areas, resulting in a very short processing time.

Due to fiber radius uniformity, variance of the maximum distance obtained in the centers is very small. For each fiber a similar peak is obtained from DT, enabling easy detection by means of thresholding. The threshold value τd is slightly smaller than the fiber's characteristic radius r (in pixels), so that τd < r.

As a result of this operation, closed regions corresponding to the fiber area are obtained, which can be located through the labeling of regions and the detection of their geometric centers. These geometric centers will constitute the feature point coordinates (non-subpixelic) to be registered in the LUT. An optimization process can be added to the detection algorithm in order to obtain subpixelic precision, although this is unnecessary since intensity changes to the fiber's core are not significant in the proximity of the center.

Let's now define the principles of our methodology, which is based on the previous FDDT calibration and a multi-fiber line-scanning calibration.

## 3. Calibration Process

Before calibration can be carried out, it is necessary to locate fibers using FDDT. These coordinates predefine where the information crucial to reordering can be found.

The calibration process consists in filling a LUT using progressive exposure of known pattern images onto the input of the bundle, and decoding the information extracted from captured images by sensor. In order to carry out this task, it is necessary to excite each fiber at the input, and verify which fiber locations are sufficiently illuminated using imaging processing techniques. The procedure can be subdivided into three steps: vertical and horizontal line scanning, the former αi estimation, and LUT calculation.

### 3.1. Vertical and Horizontal Line Scanning

**Figure 2.**
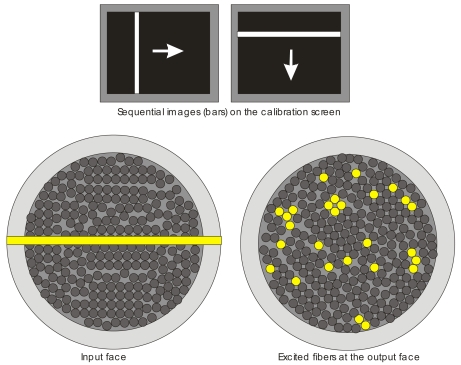
Pattern images and the effect on the bundle. Sequential images (bars) on the calibration screen. Projection at the input face. Excited fibers at the output face.

Each image comprises a fringe or bar of a given width and orientation which sweeps the input face of the bundle, first in a horizontal direction and then in a vertical direction (Figure 2). As described above, during each step of the sweeping process, the number of fibers illuminated depends on the degree of fringe and fiber input face overlap, and the particular transference function.

Sequential images are captured both in the horizontal and the vertical sweep. From these images it is possible to formulate a correspondence relationship between any excited input coordinate (i.e., row and column of a particular fringe), and any fiber location (previously determined) at the output face of the bundle which has been notably illuminated.

The width w of a projected strip must satisfy the following condition:

(6) $${\text{d}}_{\text{fib}}\geq \text{w}\geq {\text{w}}_{\text{min}}$$

where d_fib_ mean fiber diameter and w_min_ value refers to the minimal width of the projected fringe on the input face of the bundle that guarantee enough energy at the output side of the fibers.

This working range guarantees that the response of any illuminated fiber can be distinguished from the background and at the same time can be obtained only from a few relative positions. Also, a width of the projected bar greater than d_fib_ does not guarantee greater excitation because the radiance R_i_ that a particular fiber can transmit depends directly on the degree of superposition of the fringe on the fiber, rather than on width.

(7) $${\text{R}}_{\text{i}}:\Leftrightarrow {\text{A}}_{{\text{fib}}_{\text{i}}}\cap {\text{W}}_{{\text{f}}_{\text{k}}}$$

where A_fibi_ represents the area of the fiber and W_fk_ is the width of the projected fringe on the fiber in the *k* step.

**Figure 3.**
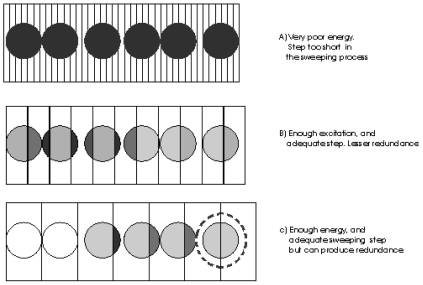
Scanning effect on the fibers.

Figure 3 illustrates some examples. Each fiber portion exposed to a fringe of a given thickness is represented by a gray level denoting the energy that can be transmitted in the entire fiber at each step. Where a fringe is too narrow, the camera may register low excitation (Figure 3a). In Figure 3b, an intermediate step is used. In this case, it is easier to determine in which position, a fiber is excited best.

The last case is similar to the second one, but the thickness of the fringe is similar to the fiber diameter. In this case, the energy of the fibers may be higher and the number of steps is reduced, but it is more probable that in two (or more) positions, the fibers receive the same excitation (ambiguous cases). This situation has been represented in Figure 3c where the last fiber is surrounded by a circle.

The higher the impact area, the greater the fiber response it is possible to achieve. Of course, if the fringe is larger than a mean diameter, it does not mean that a higher response can be attained, which is why it is advisable to work with a fringe of about ¾ of the mean fiber diameter, in order to reduce the number of ambiguous cases.

The size of the final reconstructed image is related to the number of steps required to sweep the entrance, and consequently, to the number of fibers in both dimensions. More steps diminish the number of ambiguous cases, but also produce greater separation between the points comprising the primitive image (without any interpolation), and the number of empty pixels in the image (unassigned pixels) rises considerably. Because of this, if we need to reconstruct the image, a strong interpolation process (inpainting) is required in order to refill the empty pixels. Thus, in order to select a suitable configuration, it is advisable to implement the following:

1. Estimate the maximum number of fibers in both the horizontal and vertical dimensions. This quantity is approximate to the ratio:
  (8) $$FN_{\max}=\frac{B_{\text{diam}}}{F_{\text{diam}}}$$
  where *B_diam_* is bundle diameter and *F_diam_* is mean fiber diameter. This is an approximate value, because we consider that fibers are perfectly aligned. Usually, this value is not provided by manufacturers.
2. The resolution of the camera should be sufficient to detect seven or eight pixels per fiber of diameter magnitude. This quantity enables circular elements (the fibers) in the bundles image to be identified using FDDT. Estimate the nominal fiber diameter from a bundle image (in pixel).
3. The step parameter and the fringe width on the screen should be ¾ of the mean value of the nominal diameter.

Moreover, before performing the calibration it is necessary to ensure the following:

4. The IOFB must be perpendicular to the center of the screen to minimize perspective error.
5. The input optic field of view should be slightly smaller than the active area of the monitor. This can be achieved by illuminating the entire screen with a white image and verifying that all of the fibers are illuminated.
6. The optical input should be focused on the screen, placed at about 50 cm.
7. Through the projection of the bar onto the center of the screen, it is possible to determine whether there are fibers present which are sufficiently excited to ensure a proper analysis.

Once the above tasks have been completed, the system proceeds to carry out the sweep and to fill the LUT. The structure of the LUT is indicated in Table I. It includes a parameter (α_i_) to equalize the individual responses so that, when all the fibers are illuminated homogeneously, the resulting image will present an almost homogeneous response.

Thus, the first elements incorporated into the LUT are r_i_/c_i_. The parameters R_i_/C_i_ and α_i_, are inserted subsequently, as an analysis of the entirety of images captured in the sweep is required in order to determine correspondence and best αi.

### 3.2. The First αi Estimation

In the previous task, each captured image was recorded for posterior analysis. Prior to LUT calculations, fiber responses should be compensated so that each of them has equal importance during analysis. Consequently, before this process can be carried out, a compensation factor per fiber α_i_ must be calculated and inserted into the LUT. From each captured image and fiber, a preliminary α_i_ value is calculated. Whenever the stored α_i_ value is lesser than the actual value, the data is updated. Because the fringes are white and the background is dark, the minimum value of α_i_ is reached when the fringe is nearest to the fiber center. The estimation of this parameter must be verified for both vertical and horizontal sweeps:

(9) $$\alpha_{\iota }=\min\{\frac{Gl_{\max}}{Gl_{i}}\}$$

where:

Gl_max_—Maximum gray level for the camera. For images of eight bits, this is 255.
Gl_i_—Mean gray value in the proximity (*kxk* window) of the fiber centroid.

These values are only valid in the LUT calculation. They must be recalculated at the end of the calibration process for the posterior image reconstruction process. This calculation is both simpler and quicker, requiring only an image of the IOFB when it is exposed to a white screen without saturation effect. The higher gray level provided for a fiber must be, for example, approximately 255 for an 8 bit camera. The calculation is analogous to the above described formulation.

### 3.3. LUT Calculation

Because of the intentionally high contrast of the pattern images, for any pattern image change the state of each fiber is verified by looking for which of them are sufficiently excited. When this happens, the level of excitation can also be verified by looking for the optimal position of the fringe for attaining maximum excitation. In this way the LUT is updated until all the possible positions in the entrance have been verified, compensating for the disparity of fiber response by means of α_i_.

In order to find the excited coordinates in the captured image, the stored positions (*r_i_,c_i_*) are verified for each image. The compensated gray level transmitted by each *i* fiber is calculated using a *kxk* window using an expression similar to (1):

(10) $${\text{g}}_{\text{i}}=\frac{\text{1}}{\text{k}\times \text{k}}\sum_{\text{x}=x_{ci}-\frac{k-1}{2}}^{{\text{x}}_{\text{ci}}+\frac{k-1}{2}}\sum_{\text{y}={\text{y}}_{\text{ci}}-\frac{k-1}{2}}^{{\text{y}}_{\text{ci}}+\frac{k-1}{2}}\text{I}(\text{x},\text{y})\alpha_{\text{i}}$$

A two dimensional analysis is required in order to assign the input (*R_i_,C_i_*) to a (*r_i_,c_i_*) on LUT. It is necessary to determine in which locations on the strip (*R_i_* and *C_i_*), a fiber can be illuminated and, subsequently, to determine which of them (only a pair (*R_i_,C_i_*)) is optimal for excitation. For this reason, two temporal columns are added to the LUT during calibration. These columns are necessary in order to store the highest compensated gray level for both dimensions, as a progressive description of fiber excitation. This enables the current position of each fiber to be compared with a previous, favourable position.

Firstly, for each image captured on the vertical dimension, fiber positions are scanned. Those fibers exceeding a given threshold value of gray level are considered excited. Then, the LUT is checked for previous values higher than the current response. If the current response is the highest, then the LUT is updated with the new position. This sequential process is repeated for both the horizontal and the vertical scanning.

Once the LUT has been filled, the redundancies in the stored results should be analysed. This is necessary because there are some fiber positions which could equally be allocated to different pairs of row-column. These redundancies appear when the situation described in Figure 3c, or similar, occurs and must, therefore, be eliminated.

Another case which may arise is the impossibility of assigning row and/or column values to a fiber location. This occurs when a “fake fiber position” is registered on LUT from the detection obtained from FDDT. Consequently, it cannot be assigned to any position simply because the fiber does not exist.

**Figure 4.**
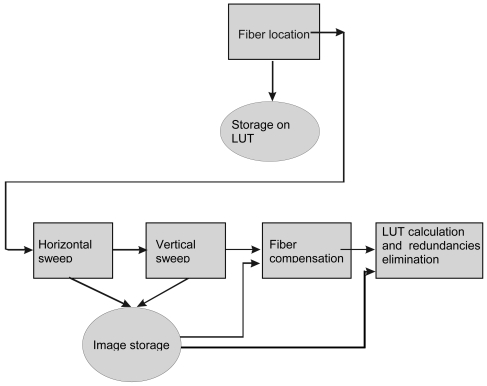
Calibration Procedure.

Lastly, the values of alpha must be updated to allow for compensation when reconstructing the images. For this procedure, rather than using images comprising stripes, a simple white image is employed. It is necessary to ensure that the captured image on the sensor is not saturated. Following this, αi coefficients are calculated using (9) and the assigned values on LUT are updated. Figure 4 shows a general procedure for calibration including all the above mentioned tasks.

## 4. Image Reconstruction—The Primitive Image

Once the LUT has been calculated, it is possible to reconstruct the image. This is an inverse procedure using data stored on LUT. A first approximation to the reordered image is the primitive image. This image is obtained directly from the g_i_ values calculated from (10), and from reordering the information provided by fibers on a 2D array whose size depends on the numbers of steps used in calibration. The new assigned positions are determined for the pair (*R_i_,C_i_*).

The image thus created may present several holes due to the non-uniform distribution of fibers at the entrance. Although this is a limitation of this image transmission method, it can be overcome through the use of inpainting techniques. Using a standard PC or a control and processing system based on FPGAs, image reconstruction can be accomplished quickly even when the process includes inpainting algorithms.

Nevertheless, the final results depend on the stability of the position of the bundle in front of the camera. Any minimal misalignment with the previously calibrated position will result in the loss of the image reconstruction. Thus, mechanical design is very important in this respect.

## 5. Results

All the results we present here were obtained with an application developed using a Matlab 7.1 simulator on a Pentium Core 2 Duo with 4 GB RAM configuration. The CMOS camera used was a BCi4-6600 manufactured by C-Can Technologies. It is a camera with linear response, 6.6 Megapixel sensor and a matrix of 2,208 × 3,000 pixels.

The fiber location procedure using FDDT was implemented. This allowed us a feasible and relatively quick means to locate most of the fibers on the IOFB. Figure 2 shows the method applied on a bundle which diameter and length are respectively 0.5 and 72 inches. The diameter of each fibre is 0.002 inches. The specific characteristics can be verified in [12]. The fibers bundle used in this test was manufactured for light guide uses. In future experiments, the bundle used will be compound by fibers destined to communications, because it presents lower attenuation, and with lengths of hundreds of meters. Table 2 shows the performance of the method.

The screen used for calibration was a 17″ with maximum resolution of 1,280 × 1,024. Given these bundle features, the maximum number of fibers is approximately 254 fibers in both dimensions. Therefore, the fringe thickness used was 3 pixels and the number of steps was 342.

**Figure 5.**
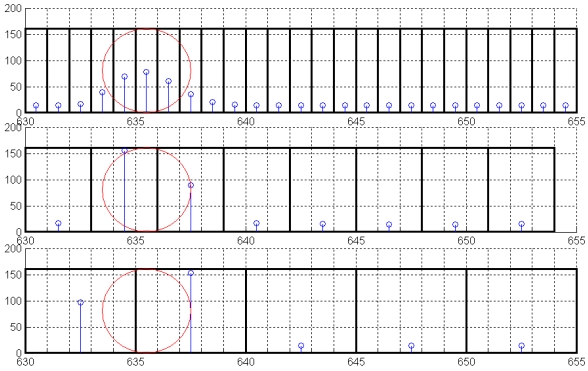
Fiber response example depending on fiber covering and fringe step.

Figure 5 shows the normalized fiber radiance response for the same fiber during each step of calibration. A different step size response is represented for a scanning line of 1, 3 and 5 pixels wide. Squares represent the area occupied by the fringe with respect to the same fiber (represented by a circle).

For the first case (1 pixel wide), the energy reached was lower than in the other cases. This is because the area that received the stripe on the circle was very small in relation to the fiber diameter. The lower the impact area, the lower the radiance at the output side. Nevertheless, if the impact area on the fiber covers an area greater than one fiber (as in the last case), then the irradiance which influences the fiber is bounded by the input face of the fiber. This situation limits the influence of the fringe on fiber radiance, at the output side.

The second case represents a fringe width of approximately 3/4 of fiber diameter. This is an acceptable value for the calibration process, showing a suitable level of excitation and a lower frequency of indeterminate cases. An initial analysis of time taken during each stage of the calibration procedure is summarized in Table 3

**Figure 6.**
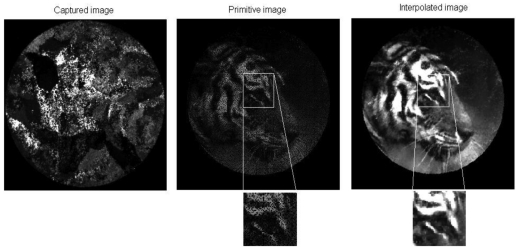
Image Progression and details.

A brief series of images of a tiger is given in Figure 6, showing the progressive transformation of the original image into the finally reconstructed image. The primitive image is obtained by rearranging the values of gray levels obtained from the fiber centroids in the bundle image, as set out in the LUT. The primitive image has about 342 × 342 pixels defined by the scanning step used. It takes about a second to reorder the information.

## 6. Conclusions

This paper presents a methodology which enables IOFBs to be calibrated for the transmission of images from remotes sites where the use of standard electronic cameras is impossible or inadvisable. Calibration permits the final reconstruction of any image projected onto the entrance of the bundle. The system described requires a very simple setup.

FDDT algorithms were implemented in order to detect the position of incoherent bundle fibers, together with a CMOS sensor on the output side. FDDT is a valid solution to the problem of fiber location, combining speed with good detection rates, fewer segmentation requirements, and low memory requirements. FDDT is rapid because the process is carried out on a binary image with small closed areas (not highly dispersed) which are clustered into a circular shape (the bundle), and thus processing time is very short.

The introduction of FDDT into the calibration process achieves a remarkable reduction in calibration time. This reduction is directly related to an important drop in LUT registers and the number of iterations needed to reconstruct images. Furthermore, it enables a very easy compensation factor calculation to be introduced into the LUT prior to calibration, and consequently, all fiber responses can be compensated for the calibration. Following calibration, these α_i_ are recalculated in a similar way, using only one image. This correction yields higher quality in the reconstructed images, with corrected intensity.

A solution to the compensation of fiber responses has been described. This problem is crucial for minimizing IOFB manufacturing defects, the effects on the edge produced by the optical input, and the manner in which the rays penetrate the same.

The IOFB used for this research was not optimized for imaging, comprising instead a light guide for illumination purposes; nevertheless the images obtained present an acceptable quality even when the primitive image is completely unintelligible.

The complete calibration process takes about ¾ hours, which is a highly acceptable timing, given that this process is carried out only once as the bundle faces remain the same as long as the position of their receptacle is not changed. Unfortunately, the authors are not aware of any previous findings in other studies which would allow a comparison between these methods to be carried out.

The time used in image reconstruction depends on the step used for calibration as well as the size of the image obtained. But in all the cases analyzed here, it comprised no more than a few seconds. These results can be optimized using a better platform of development and using a compiled version of the processing algorithms.

In future research, an analysis of this calibration technique will be implemented using different bundles with different lengths and cross sections, as well as, different materials and manufacturing technologies.

## Figures and Tables

**Table 1.** Structure of the LUT.

**Primitive LUT elements**					
	**r_i_**	**c_i_**	**R_i_**	**C_i_**	**α_i_**
**r_i_/c_i_**	**Coordinate pairs of the fibers in images captured by CMOS sensor.**				
**R_i_/C_i_**	Values describing the position of the line in the scanning process where the fiber *i*, attained maximum intensity. Each pair corresponds with r_i_/c_i_				
**α_i_**	Compensation/equalization parameter obtained to homogenize fiber intensity response				

**Table 2.** Fiber detection results.

**Number of detected fibers**	**Approximate number of fibers**	**Processing Time (min)**
49,116	50,000	≈ 0.27

**Table 3.** Calibration results.

	**Step=3**	**Step=4**	**Step=5**
Line scanning and image storage	9.14 min.	6.85 min.	5.47 min
First α_i_ calculation	7.92 min.	5.92 min.	3.2 min
Second α_i_ calculation	0.05 min.	0.05 min.	0.05 min.
LUT Calculation and verification	23.41 min.	17.6 min.	14.8 min.
Total time	40.52 min.	30.42 min.	23.52 min.
Image size	342×342	256×256	205×205
N° of redundant positions	1991	13,611	22,914
N° of points in primitive image	47,125	35,505	24,211
Primitive image calculation time with gray level compensation	≈ 1.08 s.	≈ 0.98 s.	≈ 0.55 s.
Inpainted image calculation time	≈2.17 sec.	≈2.03 sec.	≈1.35 sec.
